# Well‐Being, Pain and the Mere‐Difference View of Disability

**DOI:** 10.1111/bioe.70014

**Published:** 2025-06-25

**Authors:** James Forsdyke

**Affiliations:** ^1^ Uehiro Oxford Institute University of Oxford Oxford United Kingdom; ^2^ Campion Hall University of Oxford Oxford United Kingdom

**Keywords:** disability, identity, mere‐difference, reductionism, well‐being

## Abstract

I shall initially be discussing the term ‘disability’ in accordance with common‐sense intuition. The term itself is contested. But importantly for our discussion, on the mere‐difference view, Barnes attempts to philosophically define disability in conformity with what we ordinarily perceive to be disability day to day, and she does so by appealing to the rules of solidarity employed by the disability rights movement as definitive of those conditions that the movement should promote justice for and thus of who counts as disabled. I will operate on the assumption that this is correct during much of the essay, so as to assess whether Barnes' mere‐difference view is vulnerable to an argument from pain. I suggest that Barnes could follow three lines of argument in order to try to circumvent these difficulties, but that each of these faces further problems. I argue that for certain disabilities, in specific cases, the mere‐difference view cannot apply, particularly because pain is not necessarily sufficiently balanced by positives. Consequently, I discuss the nuances of when a mere‐difference view may be helpful, as opposed to when it might be misguided in its application, and conclude that speaking of *disability as a whole* as mere‐difference or otherwise is misguided, unless we are to reshape the concept of disability into something less heterogeneous.

## 1 Outlining the Debate

A great deal of philosophical literature has been devoted to the subject of disability, and the origins of this scholarship appear to stem from the disability rights movement. An important shift in the way that scholars talked about disability began with an organisation in Britain known as the Union of the Physically Impaired Against Segregation. As its fundamental principles explain:

In our view, it is society which disables physically impaired people. Disability is something imposed on top of our impairments, by the way we are unnecessarily isolated and excluded from full participation in society.

[1]

The social model, as it became known, has been discussed and critiqued extensively, but it led to a different way of thinking about disability, which has been drawn upon by many further scholars. The most influential of these more recent theories of disability is arguably the mere‐difference view, developed by Elizabeth Barnes in response to what she felt was a lack of understanding about the issue within philosophy. In the preface to her book, Barnes cites work by Peter Singer and Derek Parfit as particularly disheartening in this regard. She says, ‘To say that I was disappointed would be a massive understatement. “Heartbroken” might be closer to the truth’ [2].

Barnes goes on to define as disabilities those physical conditions for which the ‘rules for making judgements about solidarity employed by the disability rights movement’ apply, because they are among the things that the disability rights movement is ‘seeking to promote justice for’ [2, p. 46]. After a great deal of reasoning that relates to comparative benefits and disadvantages, she ultimately concludes that disabilities so defined are value‐neutral characteristics.

While I think that the issue of disability may be fruitful in ethical conversations, I am sceptical as to whether Barnes has really provided a satisfactory framework for understanding disability herself. In this essay, I shall first outline the mere‐difference view that she advocates for and I shall then present some problems for her explanation that derive from certain disabilities' propensity to cause pain. I then present three possible responses that would be consistent with Barnes' argument and that may show promise in favour of mere‐difference. I argue that none of these options is successful, and that Barnes draws too broad a generalisation from her initially very context‐dependent account. I suggest that reductionism about disability is a more accurate response, given her assumptions.

## 2 The Mere‐Difference Hypothesis

Here, I shall outline the hypothesis in question, and then, I shall draw out the relevance of pain as a potential counterexample. The mere‐difference theory of disability states that disability is not something that in and of itself lowers your well‐being. A variety of factors are said to aggregate, such that for some individuals, at certain times, disability may either contribute positively or negatively to their overall well‐being. For instance, having a disability in a highly prejudicial society might engender a well‐being reduction, but such effects would be the result of treatment of disability within society and not necessarily the disability itself. Barnes parallels disability with homosexuality, arguing that even if there are locally bad elements to being gay, even away from social prejudice, such as an inability to reproduce with one's partner, this does not make homosexuality value‐negative, and similarly, that therefore any pain associated with disability might be a local bad that does not necessarily bring disability as a whole into the category of bad‐difference [2, pp. 88–89]. Similarly, she identifies painful or uncomfortable aspects of being female, another nevertheless value‐neutral characteristic [2, p. 105]. She therefore refutes the decisiveness of the objection that identifies this association between disability and pain as indicative of disanalogy. She asserts that some people are better off with disability, and some do not take to it very well, and for them, it reduces their welfare, but it depends upon external factors as well as factors internal and specific to that particular person. For some, a particular disability like blindness may be construed as having a positive impact on somebody's life, the example Barnes uses being the resultant lack of need for self‐consciousness [2, p. 95]. As such, disability is value‐neutral. We cannot predetermine its case‐by‐case value.

But blindness is not painful, and so if the mere‐difference advocate wishes to generalise her claim to disability as a general category, she must show that it holds true in these cases too, in which disability seems conducive to pain. While being female may lead to certain pains, these pains are not themselves intrinsic to, or defining features of being female. But some people experience chronic pain, and that pain itself defines what it is for them to have a disability. So, it appears to me that to uphold the equivalence claim between disability more generally, in all of its forms, and value‐neutral characteristics as sex or sexuality, the mere‐difference advocate must show that the case of chronic pain does not trouble her account, and accordingly, it seems to me that such an advocate may pursue three argumentative options. The first states that pain itself can be a part of somebody's identity, and something that disabled people value, such that without it, one would be made worse off. I call this the ‘identity argument’. The second claim involves theoretically separating disability from pain, arguing that the pain that is caused by the disability may well be bad, but that this does not necessarily mean that the disability itself is bad‐difference. I call this the ‘separation argument’. The third cedes the point that disabilities may sometimes be bad for some of those who have them, but argues that indeterminacy as to whether or not a given disability will ultimately be positive or negative in its effect is sufficient to justify the claim that disability generally is value‐neutral. I call this the ‘indeterminacy argument’. I discuss some of the background issues before critically examining each. Specifically I shall show why the case of disability‐associated pain is particularly troubling for the mere‐difference account, and I then present each of the three arguments in support of mere‐difference.

## 3 Pain

Here, I show that the fact that pain is associated with some instances of disability causes the mere‐difference view theoretical difficulties.

At the same time as defending the mere‐difference view, Barnes clearly places some negative value upon pain. A common objection to the mere‐difference view is that it would seem to suggest that causing disability would be morally permissible. Barnes refutes the idea that changing a person's body in this way would be permissible on her view [3]. The explanation when it comes to altering a person's characteristics before birth is a principle of noninterference; whether it be changing a foetus' genes to make the resulting person straight, gay, disabled or able‐bodied, she thinks that the cases are all equally morally wrong [3, p. 98]. When considering adults, she makes reference to ‘transition costs’ as bearing on its clear impermissibility, one of which is the causing of ‘pain’ [3, pp. 96–97]. The potentially painful process of actually becoming disabled is argued to be the factor that does the explanatory work in this case.

But in some cases, pain will be directly linked to a disability itself, and not just with transition costs. To take an extreme case, Glover discusses the case of a newborn with ‘Dystrophic epidermolysis Bullosa’ who died after 12 weeks of genetic‐disorder‐induced agony [4]. Barnes refers to Parfit's discussion surrounding the causing of better or worse‐off future people to exist [5], in which Parfit uses an example strikingly similar to that given by Glover [4]. Here, she rejects the intuition, concluding that it is not actually wrong to cause a disabled person to exist [2, p. 107]. Barnes' negative value judgement of ‘transition cost’ pain then seemingly disappears as soon as it becomes associated with the consequences of *living with* a disability.

As Kahane and Savulescu suggest [6, pp. 783–784], a theory's generating intuitively implausible consequences should give us reason to reject or reconsider it. The most common objection to mere‐difference is, in some form or another, as discussed, that to designate disability and non‐disability as equal in value might entail that either it is permissible to cause disability or that it may be impermissible to prevent it. I do not believe it necessary to even appeal to this argument in order to show that Barnes' theory is flawed. Considering Barnes' stated conclusions will be sufficient enough. Her dismissal of relevance when it comes to disability‐related pain is one such counterintuitive conclusion, as is her commitment to there never being any wrong in causing disabled infants to exist, all being equal, regardless of the disability, or its severity. Having thus shown that the issue of pain appears to generate some inconsistencies within Barnes' account, I will next consider three primary defences of the argument that she could plausibly make, showing that for each, the logic behind them is questionable and that the conclusions overgeneralise to the point of implausibility. I start with the first major line of defence consistent with her account, which I have called the identity argument.

## 4 The Identity Argument

Consider the way in which Barnes arrives at the conclusion that disability is mere‐difference. The same logic could be applied for pain. Under certain circumstances, pain may arguably be a good thing, and so generally, it might be classified similarly as neutral simpliciter. Take away pain, and we are no longer aware when we need to avoid something dangerous, like touching something hot. It has an instrumental function in certain contexts, and this could manifest in a number of other senses too. Mere‐difference proponents could consider making the claim that pain can be character‐building for disabled people.

This becomes reminiscent of the concept of soul‐making [7]. Traditionally used in theological contexts, the problem of evil, the question of how an omnibenevolent and omnipotent God could allow evils (pain) to exist, has been explained by its necessity for shaping ‘the soul’. Hardships may be tests designed to improve us and strengthen character in various ways. Some disability activists argue in the same vein that disability is merely a difference in ability, something that may create hardships in certain contexts, but that these disability‐associated hardships strengthen the character of people with disabilities in such a way that they are better off for them. An example picked up by Barnes illustrates a case in point [2, p. 115]. Here, she describes the argument presented by LaSpina. LaSpina argues that she is proud of her disability despite the chronic pain that it causes, and that this is rationally equivalent to her being proud of her Italian nationality in spite of the existence of the Italian mafia. Barnes quotes her, saying that she is happy and proud to be ‘a person with a disability—with whatever pain there is. That's part of it’ [2]. Perhaps a person with a disability has had to face challenges that others have not, such that those experiences enrich their character positively, far beyond the intrinsic badness of the pain experienced.

Let us grant that disability is not necessarily intrinsically a disadvantage, and that contextual contingency matters, as Barnes so argues. Kahane and Savulescu argue that there is another way in which something can be bad, namely, that it tends to produce more negative experiences than positive on average [6]. Disabilities, they argue, are often factors that reduce well‐being overall, and would, even discounting social prejudices. Barnes often charges arguments from such intuitions about disability as deriving from widespread ableist attitudes [2, p. 155]. But Barnes herself, in defence of her theory's compatibility with hedonism, admits that ‘pains [would] need to be correlated with pleasures’ in order for a painful disability to be value‐neutral [2, p. 116]. So, do disabilities involving chronic pain bring people benefits in equal amount to the costs?

Even if there are unique experiences to be had from being disabled, such as participation in a ‘disability culture’ [2, p. 117], it is not at all clear that ‘most disabilities are mere‐difference’ [2, p. 103]. A disabled person could have access to said disability culture if their chronic pain levels were on average a 2/10, and likewise, the same level of access if their pain's severity reached 9/10. Similarly with other unique experiences and benefits to being disabled, it seems very implausible to suggest that a disabled person's experiences of the good aspects multiply in either rough or precise concordance with the extent and severity to which one experiences negative consequences such as pain. Theoretically, we could take a given disability and ask what the effect of adding more pain would be. I have serious doubts that positive experiences would increase with every additional incremental increase in pain. I will henceforth talk of ‘unnecessary pain’ as referring to that pain that has no instrumental function or that goes above and beyond having any further character‐building utility.

The mere possibility of pain being a positive or neutral fact of life for some disabled people such as LaSpina does not make it true in all cases. Nadelhoffer provides an account of his own chronic pain that stands in direct contrast with LaSpina; in fact, he says that he would ‘pay any price for’ a treatment, even if it subtracted years from his life [8]. Clearly, chronic pain can make one's life go worse. Chronic pain can be intrinsically unpleasant, disruptive to one's life aspirations, can make a person worse off than counterfactually would be the case and is overwhelmingly bad for some people; hence, among sufferers, ‘30% struggle with suicidal ideation’ [8, p. 16].

At this point in her argument, Barnes appeals to a generalised characterisation of disabled people's experiences as positive, as evidence that pain has little influence as to whether disabled people value their disabilities [2, p. 103]. This evidently ignores some people's experiences, but even if we ignore the problem of inter‐disability generalisability, a more fundamental problem exists in her logic.

Barnes emphasises that given the existence of a ‘troubling history’ relating to minority groups not being listened to, we have a sufficient reason to take disabled people at their word in this case [9]. But arguments based upon induction have their drawbacks. A minority of people see it as absolutely necessary that they be as skinny as possible. Some people are drawn into a very unhealthy minority culture, perhaps with its own rules of solidarity and encouragement, which believes that being anything but incredibly thin is bad for you, or your image, and encourages anorexic self‐starvation. This is not in fact healthy; hence, minority groups are not guaranteed to always be right. Past experiences do not always translate well into future cases.

If Barnes were to refute the thought that these people form a minority group, then I would point to the fact that she defines disability, a supposed mere‐difference, in much the same way, as being characterised by those things that the disability community cares about promoting the culture of and justice for [2, p. 46]. Likewise, paralleling Barnes' argument that not being disabled (perhaps specifically not experiencing chronic pain) makes a person epistemically unfit to judge how badly off a person with said disability might be [2, p. 6], I could point to the fact that using this same logic would preclude judgement in the case of the people starving themselves to be thin, or indeed in other extreme cases. I do not know what it is like to be in a permanent coma, but I know that it is bad without having ‘been there’. Barnes seems to want philosophy to work differently when a minority group is involved, but we have good independent reasons to say that unnecessary pain is bad.

Otherwise, one may interpret Barnes' argument as saying that the pain of having a certain disability may feed into the person's identity, in a unique way, without which the person in question would no longer be themselves. Perhaps LaSpina is proud of who she is, in the sense of ‘all of the things which make up me’. Some of these elements might be bad for her life, but she would not go without them, on the basis that they constitute who she is at the present moment. If pain has a significant impact upon a person's character, then perhaps it would be legitimate to prefer to remain as one is.

There are two flaws in this argument. First, being alleviated of pain would not cause a drastic change in psychological continuity [5, p. 315]; the person would most likely retain any disability‐related influences on ‘character‐building’ that they had accrued throughout their life so far, and assuming that is a positive, then also being alleviated from future pain seems like a further benefit. The claim would rely upon there not being diminishing marginal returns on character‐building from future pain, and as I have established, it does not seem true to say that any benefit to being disabled would increase proportionally with the experience of further pain. Second, let us posit that someone has a preference to keep their disability‐associated pain, and even assume that this is a reasonable preference for them to have. It does not follow from having a reasonable preference for staying the same that doing so will be the best thing for them to do, nor does it follow that other people should adopt the same preferences [10], such that we could say ‘for all cases of disability, they are mere‐differences’.

Having established that the identity argument does not hold, I would like to now move on to the second: the separation argument.

## 5 The Separation Argument

Barnes' discussion of local and global harms is indicative of the second argument [2, pp. 80–81]. A characteristic is a local bad for a person iff it reduces their welfare with respect to a particular contextual factor such as time or place. A characteristic is a global bad iff it simply has a negative effect on a person's well‐being, irrespective of circumstances. Finally, a trait is bad simpliciter if its absence would counterfactually increase well‐being for any given person [2, pp. 86–67].

Given the fact that she refers to pain as a transition cost, Barnes does clearly think that pain is bad in some sense. But if we apply Barnes' logic to pain, then we can classify it as neutral simpliciter. Some cases of experiencing pain are such that removing the pain would make us worse off, for instance, in the ‘hand on the hob’ case, or certain character‐building cases. Other cases are such that the pain reduces a person's welfare, where it is unnecessary, a local bad. Likewise, Barnes' main claim is that on its own, disability is neutral simpliciter [2, p. 88]. Not every disability is such that removing it would make a person better off. An autistic person's traits might give them the ability to think in a very unique way, such that they bring about discoveries within a particular academic field (say) not previously thought possible, and hence live a happy life. A little social accommodation may make a big difference to their well‐being. With regard to disabilities that involve chronic pain, what I believe a mere‐difference proponent could say in such a case is that resultant pain is locally bad for the person, in the same way that being autistic may (perhaps sometimes) pose locally bad social communicational challenges, but that the disability that engenders it is not necessarily bad itself.

As discussed, pain conducive to character‐building seems to have diminishing marginal utility, up to the point where further pain falls within my definition of ‘unnecessary pain’. Beyond that, it makes the disability globally worse. But perhaps we could use painkillers to mitigate against unnecessary pain. If the badness of disability is dependent upon unnecessary pain, then removing the causal link between the two might help make the disability a difference as opposed to a detriment, the removal of which would not automatically constitute a life improvement.

But this only shows that some disabilities could hypothetically be brought into the realm of being contingently mere‐difference, where they would not be otherwise. This would all depend upon context. Having a plentiful supply of painkillers worldwide might thus make a chronically painful disability less of a bad quality. But of course, as things stand, chronic pain is not something that can be totally prevented.

Even the idealistic possibility of detaching the causal link between disability and unnecessary pain is not sufficient to prove that all disability is mere‐difference. First, as mentioned previously, an individual's preference for something based upon their own subjective experience does not independently make that thing something that is as good as the alternative [10, p. 28]; second, if based on Barnes' definition, Richard III was disabled [2, p. 51], then her conception of disability covers those who lived in the past and experienced chronic pain. Hence, if these too are instances of disability, some instances of disability on Barnes' account are clearly bad‐difference, particularly given the existence of overwhelming chronic pain both in the past and in the present day. No appeal to a hypothetical detachment of unnecessary pain from disability will change that.

Barnes does not rely upon this argument from social accommodation, instead claiming that her view is compatible with disability being associated with disadvantages that cannot be mitigated against by society; rather, critically, her claim is actually that there is never a systematic connection between a disability's disadvantages and a disabled person's overall well‐being [2, pp. 9–10]. But, as I have shown already, there are disabilities that predictably and reliably significantly shorten lifespan and cause unbearable chronic pain that makes sufferers' lives worse off. It would be implausible to suggest that these disabilities are mere‐differences.

As neither of these options work, a third defensive argument is necessary. This defence acknowledges that some instances of disability are bad for those who have them, but tries to circumvent this via appeal to indeterminacy.

## 6 The Indeterminacy Argument

Fundamentally, Barnes is open to the fact that the value‐neutral model could be compatible with the claim that ‘individual disabilities are in fact bad‐difference’ [2, pp. 102–103]; she simply feels uncomfortable with the thought that we might say of a particular disability that it is or is not mere‐difference, and cautions against trying to determine which are, on the basis of the inductive ‘minority group experience’ claim, that I have determined is on shaky theoretical ground.

Barnes would appeal to this indeterminacy as evidence that, when we speak about disability, we cannot determine in advance whether a case will turn out positively like LaSpina's, or something very negative like Nadelhoffer's. Here, then, lies the final defence of mere‐difference. If we cannot ‘automatically’ predetermine whether a person's having a disability will make them ‘worse off’ [2, pp. 94–95], then perhaps ‘disability’ as a cluster concept is a mere‐difference.

However, this too is vulnerable to the argument from tendencies. Winning the lottery is a good thing, even if it can sometimes make people's lives worse via conflicts or privacy feuds. If certain disabilities tend to make us worse off, then the existence of a few good experiences is not reason enough to generally ascribe value‐neutral status to them. It seems perfectly conceivable that someone might say of a particular disability: *Generally, X tends to be a bad thing, but I've found it a positive experience*.

Importantly, I think that this final argument reveals a major contradiction within Barnes' theory. Barnes generalises her claims about disability, but what makes her so sure that she can? Why does Barnes stop at this level of abstraction? As discussed, Barnes starts from a common‐sense model of disability, its broad scope defined by the ‘rules’ of ‘solidarity’ used by the disability movement [2, p. 46], and so far, I have been operating on the assumption that she is correct. Despite these common‐sense assumptions, she arrives at conclusions that are anything but common‐sense. Her logic in between has the benefit of being heavily grounded in context, but the benefit of context‐based accounts is supposed to be that they get the right results in individual cases. Given this, it is difficult to understand why Barnes did not take the logical next step and become a reductionist about disability.

## 7 Reductionism About Disability

If Barnes is correct in saying that social and environmental contexts, as well as personal contexts, matter to disability, if certain circumstances combine to determine whether or not a disability's adverse effects are balanced by positives, then the extent to which mere‐difference status holds true is likely to vary not just between different disabilities but also between different people, within separate manifestations of the same disability. If different people, depending upon their context, social, environmental and personal, formulate different opinions about how valuable or otherwise their personal manifestation of disability is to them, then why does Barnes feel the need to ignore said context and generalise her mere‐difference claim to ‘disability’ as a whole?

The fact that pain varies in quantity between disabilities, varies to different degrees even within disabilities and between different people's experience of them and varies in its value or proclivity to produce positive versus negative experiences seems to me to suggest that talk of mere‐difference applying to ‘disability’ universally [2, p. 78] is to miss the point. My claim then would be that severe and chronic pain are decisive counterexamples to the view that ‘disability’ generally speaking is *mere‐difference*, but equally not strong enough to justify labelling ‘disability’ *bad‐difference* generally speaking, so long as we take a common‐sense, reductionist view of disability. I concur with Nadelhoffer that ‘invariantism’ about the value of disability does not work [8, p. 24]. Some disabilities may be mere‐differences for some people; other disabilities, and even the very same disabilities, may be bad‐differences for other people. The mere‐difference status of a person's disability may even vary situationally. This is a far weaker claim than Barnes makes, but one that I think is nevertheless much more accurate.

One could of course redefine disability, and move away from the common‐sense cluster concept, perhaps positing that a requirement of a disability is that it significantly reduces somebody's range of abilities [11] or their welfare [12] in some way, excluding the impact of any social prejudices. This would be an interesting point of investigation; however, I will not undertake it here. It suffices for my argument to point out that, in doing this, a mere‐difference ‘disability’ would then no longer be an instance of disability; however, if we decided to do the reverse, and say that any individual case of a bad‐difference disability is not a disability, then I think that would contradict the spirit of disability activism and would be gravely unfair, particularly given that such people would be in even greater need of the justice‐oriented rights movement Barnes discusses and positions as being so important to disabled people. So, there seems no way to accurately generalise, such that ‘disability’ is universally value‐neutral. I have demonstrated this via appeal to cases of disability that involve chronic pain, where there is no comparable benefit to be derived from such pain.

## 8 Conclusion

Having tested three potential lines of defence for generalising the mere‐difference claim across the cluster of things Barnes calls ‘disabilities’, I conclude that the argument is unconvincing. It takes context to matter, and then discards this commitment when it comes to cases in which it might otherwise prove the opposite. The case of chronic pain is a case in point, where testimony is mixed, and for some, it seems that its cessation would allow people living with it to experience more fulfilling lives. Having said that, there are cases where disability, as it is commonly understood, will be a mere‐difference and I think cases where choosing to remain disabled could be an entirely reasonable or even a rational preference. It simply is not an issue that we can generalise about, and this stems from the fact that disability, as it is currently conceived of, is such a heterogeneous category.

## Data Availability

Data sharing is not applicable to this article as no new data were created or analysed in this study.
